# Polymorphism of CD14 Gene Is Associated with Adverse Outcome among Patients Suffering from Cardiovascular Disease

**DOI:** 10.1155/2021/3002439

**Published:** 2021-07-08

**Authors:** Susanne Schulz, Martin Zielske, Sascha Schneider, Britt Hofmann, Hans-Günter Schaller, Axel Schlitt, Stefan Reichert

**Affiliations:** ^1^Department of Operative Dentistry and Periodontology, Martin Luther University of Halle-Wittenberg, Germany; ^2^Department of Cardiac Surgery, Mid-German Heart Centre of the University Hospital Halle (Saale), Martin Luther University of Halle-Wittenberg, Germany; ^3^Department of Internal Medicine III, Heart Centre of the University Clinics Halle (Saale), Martin Luther University of Halle-Wittenberg, Germany; ^4^Department for Cardiology, Paracelsus Harzklinik Bad Suderode, Germany

## Abstract

**Background:**

The biological link between severe periodontitis and cardiovascular disease is well established. Both complex inflammatory diseases are influenced by genetic background. Therefore, the impact of genetic variations of receptors of the innate immune system—(Toll-like receptors (TLRs)) TLR2, TLR4, cluster of differentiation 14 (CD14), and the transcription factor nuclear factor-*κΒ* (NF-*κ*B)—was investigated.

**Materials and Methods:**

In this study (ClinicalTrials.gov identifier: NCT01045070), 1002 cardiovascular (CV) patients were included. In a 3-year follow-up period, new vascular events were assessed. SNPs in CD14 (rs2569190), NF-*κΒ* (rs28362491), TLR2 (rs5743708), and TLR4 (rs4986790) were genotyped. The impact of these genetic variants on severe periodontitis as well as on CV outcome was assessed.

**Results:**

All investigated genetic variants were not associated with preexisting CV events or severe periodontitis in CV patients. In Kaplan-Meier survival analyses, the CT genotype of CD14 single-nucleotide polymorphism (SNP) rs2569190 was shown to be an independent predictor for combined CV endpoint (log rank: *p* = 0.035; cox regression; hazard ratio: 1.572; *p* = 0.044) as well as cardiovascular death (log rank: *p* = 0.019; cox regression; hazard ratio: 1.585; *p* = 0.040) after three years of follow-up.

**Conclusions:**

SNPs in CD14, NF-*κΒ*, TLR2, and TLR4 are no risk modulators for preexisting CV events or severe periodontitis in CV patients. The CT genotype of CD14 SNP rs2569190 provides prognostic value for further CV events within 3 years of follow-up.

## 1. Introduction

The immune system consists of a multitude of innate immune receptors to recognize pathogens. Among these receptors, pattern recognition receptors (PRRs), including the Toll-like receptors (TLRs) and cluster of differentiation 14 (CD14), have long been established to play a key role in the host defense system [1, 2]. In particular, lipopolysaccharides (LPS) are recognized by TLRs, which are localised in the membranes of immune cells and are the first receptors to be activated in the interaction between pathogen and host interaction [3]. TLR2 is capable of recognizing lipoproteins and peptidoglycans from both Gram-positive and Gram-negative bacteria, as well as LPS and lipoteichoic acid from Gram-positive bacteria [4]. On the other hand, TLR4, in the interaction with the cluster of differentiation (CD14), is able to recognize LPS from Gram-negative bacteria [4]. In the course of the thereby initiated signal transduction cascade, many mediators are produced which contribute to the control of infection. Among these mediators, transcription factor nuclear factor-*κΒ* (NF-*κΒ*) is an important control element that is able to promote the immune response by releasing further proinflammatory cytokines [1].

It was shown that in addition to immune cells, cells such as fibroblasts including oral fibroblasts, epithelial cells, and endothelial cells can also contribute to the activation of innate immunity via PRRs [3, 5]. In this context, PRRs, including TLRs and CD14, as well as downstream signal transduction cascades have been implicated in pathogen recognition during periodontal infection [6]. Furthermore, activation of PRRs triggers the inflammatory response in various physiological systems, including the cardiovascular (CV) system. PRRs including downstream signal transduction cascades have been discussed as key contributors to the progression of various CV diseases including, e.g., atherosclerosis and heart failure [3].

A biologically plausible connection between periodontal and CV diseases has been assumed for many years [7]. PRRs and the downstream signal transduction cascade could be provided a biologically appropriate link between these two diseases. Among other things, a common genetic predisposition is discussed as an interface between both diseases [8]. The focus of this study will be on genetic variants of PRR candidate genes CD14, TLR2, TLR4, and NF-*κΒ* as a representative of downstream regulation. The main attention will be given to functionally important genetic variants for which an association with both diseases could be suspected. In the CD14 gene, a promoter polymorphism (rs2569190; c.-260C/T) was shown to be associated with increased transcriptional activity resulting in higher levels of soluble CD14 [9]. In the TLR4 gene, the SNP rs4986790 (Asp299Gly) was described to be associated with functional alterations that predispose individuals to respond less sensitively to LPS and to have an increased susceptibility to pathogenic bacterial infections [10–12]. SNP rs5743708 in the TLR2 gene (Arg753Gly) has been implicated in reducing the capability of TLR2 to target bacterial cell wall components [13]. In the NK-*κΒ* gene, the rs28362491 SNP represents a 4 bp ATTG insertion/deletion variation at position −94 bp of the promoter. This polymorphic site has been reported to have a major impact on gene expression, with deletion of ATTG resulting in reduced promoter activity [14]. Due to their functional consequences, all described SNPs are highly involved in the immune response and could thus represent a possible link between periodontal and cardiovascular diseases. And indeed, in a variety of case-control studies as well as in meta-analyses, their impact on periodontitis as well as CV diseases was assessed (CD14 [15–17], NF-*κΒ* [14, 18, 19], TLR2 [20, 21], and TLR4 [4, 22, 23]).

However, the significance of these SNPs in linking periodontitis and CV diseases has not been at the focus of investigations to date. Based on the data available, we assessed in our study the impact of SNPs in TLR2 (rs5743708), TLR4 (rs4986790), CD14 (rs2569190), and NF-*κΒ* (rs28362491), on severe periodontitis as well as on the incidence of new CV events within a three year follow-up in a cohort of CV patients.

## 2. Materials and Methods

### 2.1. Cohort of Cardiovascular Patients

The present investigation is a substudy of the longitudinal cohort study “Periodontitis and Coronary Heart Disease (CHD)” ClinicalTrials.gov identifier: NCT01045070. A total of 1002 coronary patients of the Department of Internal Medicine III were included in the study consecutively from October 2009 to February 2011. The inclusion and exclusion criteria for coronary patients, including periodontal and cardiovascular examinations, and determination of biochemical parameters have already been described in detail [24]. Therefore, it will only be briefly addressed here. All patients were at least 18 years old and of Caucasian descent. They have been suffering from ≥50% stenosis of a main coronary artery (angiographically proven). All patients had at least 4 of their own teeth.

The exclusion criteria applied were inability to give written informed consent, pregnancy, subgingival scaling, root planning during the last 6 months, antibiotic therapy during the last 3 months, current alcohol or drug abuse, and medication of drugs that potentially cause gingival hyperplasia (e.g., hydantoin, nifedipine, and cyclosporin A).

All patients underwent a standardized cardiovascular and dental examination and were evaluated for biochemical peculiarities. Regarding cardiovascular parameters, the left ventricular ejection fraction was assessed and serum parameters including haemoglobin, interleukin-6, C-reactive protein, low-density lipoprotein, high-density lipoprotein, triglycerides, and creatinine were measured. The dental examination comprised of determining the plaque index and bleeding on probing after 30 seconds at four sites around each tooth. Both maximal clinical probing depth (PD = distance between gingival margin and the bottom of the pocket) and maximum clinical attachment loss (CAL = distance between the cementoenamel junction and the bottom of the pocket) were taken at six sites around each tooth applying a pressure-sensitive dental probe UNC 15 (0.2N) (Aesculap, Tuttlingen, Germany). For calculating the mean values, the maximum values for each tooth were considered.

According to the periodontitis classification of Tonetti and Claffey, the coronary patients were evaluated regarding their severity of periodontitis [25]. A severe periodontitis case was diagnosed if ≥30% of the teeth that were present showed a proximal attachment loss of ≥5 mm. A periodontitis case was considered as the presence of proximal attachment loss of ≥3 mm in ≥2 nonadjacent teeth. All remaining coronary patients exhibit no periodontitis.

After 3 years, a follow-up was performed from November 2013 until January 2015. Data regarding the predefined combined endpoint (myocardial infarction, stroke/transient ischemic attack (TIA), cardiac death, and death caused by stroke) were collected. Using a standardized questionnaire, the patients (or relatives or patient's physicians) were interviewed by mail or phone. If follow-up information could not be obtained from these persons, civil registration offices were contacted and information about current address or date of death was requested.

### 2.2. Genetic Investigations

The genomic DNA was isolated from leucocytes of venous blood using QIAamp® blood extraction kit (QIAGEN, Hilden, Germany) in accordance with the manufacturer's manual.

For genotyping, specific restriction fragment length polymorphism (RFLP) analyses were established for all SNPs (Table 1).

### 2.3. Statistical Analyses

Statistical analyses were carried out using the SPSS software 25.0 (SPSS Inc., Chicago, Illinois). Values of *p* < 0.05 were considered to be significant. Metric data were tested for normal distribution using the Kolmogorov-Smirnov test. Categorical variables were documented as percentage and plotted in contingency tables and evaluated using the chi-square test and Yates continuity correction. If the expected cell frequency was <5, Fisher's exact test was applied.

For survival, evaluation Kaplan-Meier analyses with log-rank tests were applied. Confounding effects on survival (adjusted hazard ratios) were investigated using Cox regression.

## 3. Results

In the present longitudinal cohort study, 1002 patients were prospectively involved. Clinical characteristics of the patients are displayed in Table 2. 953 individuals could be evaluated in the 3-year follow-up (follow-up time: 152.9 ± 49.5 weeks). This corresponded to a dropout rate of 4.9% after three years of follow-up. The total incidence of the combined endpoint was 16.4% (stroke/TIA: *n* = 23, myocardial infarction: *n* = 33, cardiovascular death: *n* = 93, and death from stroke: *n* = 7).

### 3.1. Possible Association of the Genetic Background and the Prevalence of Periodontitis

Possible associations between SNPs in CD14 (rs2569190), NF-*κ*B (rs28362491), TLR2 (rs5743708), and TLR4 (rs4986790) and the occurrence of severe periodontitis were investigated in the cohort of CV patients. Codominant, dominant, recessive genetic models for all SNPs were tested. In addition, possible associations between allele distribution and severe periodontitis were also included in the assessment. Here, we could not demonstrate any significant associations between genetic markers and the severity of periodontitis (Table 3).

### 3.2. Genetic Characteristics and the Cardiovascular Prognosis in the 3-Year Follow-Up

**Figure 1:**
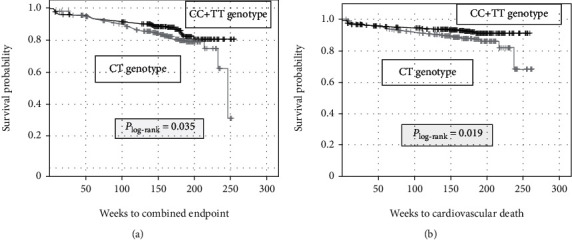
Kaplan-Meier plot for the incidence of the (a) combined endpoint (stroke/TIA, myocardial infarction, cardiovascular death, and death from stroke) and (b) cardiovascular death within a 3-year follow-up period according genotype distribution of CD14 SNP rs2569190. Statistical comparison was made by the log-rank test.

The impact of SNPs in CD14 (rs2569190), NF-*κΒ* (rs28362491), TLR2 (rs5743708), and TLR4 (rs4986790) on the incidence of combined endpoint defined as CV death, death from stroke, MI, and stroke/TIA was evaluated. In bivariate analyses without taking survival time into account, neither the genotype nor the allele distribution was shown to be associated with the adverse CV outcome (Table 4). However, when survival time as well as further genetic models were considered, a different conclusion was obtained. After Kaplan-Meier analysis and log-rank test, the CT genotype of rs2569190 in the CD14 gene was as a prognostic marker for the combined endpoint (*p* = 0.035) (Figure 1(a)). Carriers of the CT genotype had a considerably poorer prognosis than CC + TT genotype carriers (incidence of combined endpoint: CT: 18.9% vs. CC + TT 13.5%, *p* = 0.036). Taking established cardiovascular risk factors, including increasing age, male gender, body mass index, diabetes mellitus, hypertension, and hypercholesterolemia, as well as severe periodontitis into consideration, the CT genotype was proven as an independent risk modulator of the combined endpoint (HR = 1.572, *p* = 0.044) (Table 5(a)). A detailed examination of the CV endpoint showed that the CT genotype had a particular impact on CV death (Kaplan-Meier analysis including log-rank test: *p* = 0.019, Figure 1(b)). Considering further CV risk markers in Cox regression analysis, the hazard ratio for the CT genotype measured 1.585 (*p* = 0.040) (Table 5(b)).

In this study, increasing age (HR = 1.063, *p* < 0.001) and diabetes mellitus (HR = 2.366, *p* < 0.001) were shown to be additional significant predictors for adverse cardiovascular events (combined endpoint and cardiovascular death (Table 5).

All other genetic combinations of the SNPs investigated in the study did not show prognostic value for combined endpoint.

## 4. Discussion

The focus of this longitudinal cohort study was to examine the influence of SNPs in PRR genes and a gene of the downstream signal transduction cascade on severe periodontitis and to assess their prognostic significance for future CV events in patients with angiographically proven significant stenosis.

### 4.1. SNPs and Severe Periodontitis

In this cohort of CV patients, no significant associations between SNPs in CD14 (rs2569190), NF-*κΒ* (rs28362491), TLR2 (rs5743708), and TLR4 (rs4986790) and the prevalence of severe periodontitis was proven considering codominant, dominant, and recessive genetic models as well as allele distribution. The studies conducted so far on the possible association of genetic variants in PRRs as well as NF-*κ*B and periodontitis are very heterogeneous.

Considering SNP rs2569190 in the CD14 gene, an association of this SNP with periodontitis could be shown in highly selected case-control studies [26–28]. But in contrast, other case-control studies did not confirm this association [29, 30]. However, in meta-analyses, no association of rs2569190 in the CD14 gene with periodontitis could be confirmed [15, 31]. Furthermore, applying stratified analysis by ethnicity and the severity of periodontitis, no significant associations were assessed between CD14 SNP rs2569190 and periodontitis [15, 31]. Investigating the impact of these SNPs on the occurrence of aggressive or chronic periodontitis, a meta-analysis was performed [17]. And again, no significant genetic impact could be evaluated.

Although the importance of NF-*κΒ* in the immune response is undisputed, only few case-control studies have been conducted to investigate possible association of the SNP rs28362491 with periodontitis. The study situation is very inconsistent. Whereas, for example, Toker et al. [19] could not show any association of the SNP with periodontitis and Schulz et al. [18] demonstrated that del/del genotype carriers suffered more frequently from aggressive periodontitis.

Recently, case-control studies on the significance of the SNP rs5743708 in the TLR2 gene in periodontitis were summarized in a meta-analysis [20]. In this analysis, no association of this SNP and periodontitis, including disease-specific subgroup analysis (aggressive, chronic periodontitis), was confirmed. However, ethnic stratification revealed an indication that the SNP is associated with periodontitis in Asians [32].

Regarding rs4986790 in the TLR4 gene, the study situation is also heterogeneous. However, it should be emphasized that meta-analyses have not confirmed the association of this SNP in the TLR4 gene with periodontitis, even after stratification for ethnicity and disease severity [31, 33]. In contrast, in a meta-analysis by Chrzęszczyk et al. [34], this SNP was shown to be in association with chronic periodontitis. Furthermore, specific studies have shown that this genetic variant is associated with the occurrence of periodontitis in male [4] and with periodontitis in the presence of *P. gingivalis* infection [35].

All the meta-analyses or case-control studies presented so far have in common that no study evaluated possible associations between these genetic variants and periodontal diseases in a cohort of CV patients. Since periodontitis as a chronic inflammatory disease is characterized by a complex interaction of different mechanisms, factors interrelating with the immune system (e.g., cardiovascular diseases) must be considered as confounding modulators [7]. For this reason, patients of the present study might be particularly predisposed for periodontal inflammation due to their existing CV disease. Consequently, the current genetic results cannot be compared with the studies presented.

### 4.2. SNPs and the Cardiovascular Prognosis in the 3-Year Follow-Up

Because of their important role in CV disease, we hypothesized that SNPs in PRRs (CD14, TLR2, and TLR4 gene) and the NF-*κΒ* gene might also have a prognostic value for further CV events.

SNP rs2569190 located in the promoter of the CD14 gene has been shown to modulate inflammatory stimulation by regulating CD14 gene expression and the concentration of soluble CD14 (sCD14) in plasma [36]. Because of these functional effects in the context of the immune response, the SNP has been attributed an important role in inflammatory diseases.

In a genome-wide association study, the CD14 locus was shown to be associated with CV disease [37]. In particular, the SNP rs2569190 was related to the occurrence of CV diseases in case-control studies as well as meta-analysis [16, 38, 39]. In meta-analyses, the TT genotype and T allele of rs2569190 have been associated with ischemic heart diseases [38] and the susceptibility and development of cardiovascular disease [16], particularly in the East Asian population but not European population. In accordance with these studies, we also did not demonstrate a genetic influence of CD14 SNP rs2569190 on the incidence of cardiovascular preexisting conditions (stroke, myocardial infarction, and peripheral arterial disease) in the present European cohort of CV patients.

However, the focus of the present study should be on the impact of SNPs on CV prognosis. In the presented study, the CT genotype of CD14 SNP rs2569190 was demonstrated to be associated with an adverse CV outcome in relation to the combined CV endpoint (log-rank: *p* = 0.035; cox regression; hazard ratio: 1.572; *p* = 0.044) as well as cardiovascular death (log-rank: *p* = 0.019; cox regression; hazard ratio: 1.585; *p* = 0.040) after three years of follow-up.

So far, only few data are known about a possible prognostic significance of the SNP rs2569190 with regard to the cardiovascular outcome. In a study by Porsch-Ozcürümez et al., T allele carriers were shown to have a 3.6-fold higher risk for nonlethal CV events (log-rank test = 0.029) in a 4-year follow-up period [40]. In contrast, in the present study, the TT genotype or T allele was not proven to be independent predictors for CV prognosis. Although the inclusion criteria of this study were comparable to those of the present study (≥50% stenosis of a main coronary artery; CV patients of a similar geographical region: Central Germany, Saxony-Anhalt), the CV endpoints differed. While Porsch-Ozcürümez et al. defined nonlethal CV events as endpoints in the present study, cardiovascular death and death from stroke were also included as endpoints. Furthermore, the enrolled patients in the present study were *n* = 1002, which is significantly higher than the number of patients in the study of Porsch-Ozcürümez et al. (*n* = 69).

SNP rs28362491 in the ubiquitous transcription factor NF-*κΒ* was reported to be involved in the expression of immune-modulating genes due to its impact on NF-*κΒ* gene transcription [41]. Therefore, this SNP has been implicated in inflammatory diseases including cardiovascular diseases [14]. In the meta-analysis by Wang et al., the deletion allele was shown to be associated with CV diseases even after stratification by ethnicity and gender [14]. However, a corresponding association could not be confirmed in the present study in the cohort of CV patients. The SNPs including all genetic models were independent of stroke, myocardial infarction, and peripheral arterial disease. However, when comparing the studies, it is important to note that the present study only included patients with a history of cardiovascular disease (inclusion criterion: ≥50% stenosis of a main coronary artery). Furthermore, it was shown that this SNP has no predictive power for an adverse CV outcome in the present study. As this issue has not been addressed in previous studies, no comparisons with the existing evidence can be provided here.

TLR2 is involved immune response due to its recognition of many pathogen-associated molecular patterns. The SNP rs5743708 has been described to have immediate functional significance regarding altered signaling [42] and might be implicated in inflammatory diseases [21]. A few studies were conducted in order to evaluate the possible association between this SNP and CV diseases; however, results are controversial. Whereas Golovkin et al. did not reveal a genetic influence on infective endocarditis [21], Guven et al. reported on an association with CV susceptibility in Turkish patients [43]. However, in the present study, an influence on cardiovascular burden (stroke, myocardial infarction, and peripheral arterial disease) could not be confirmed in the cohort of CV patients of Central Germany. Since the inclusion criteria of the present study were comparable to those of Guven et al., ethnic characteristics could possibly play a role in the discrepancy of the results.

With regard to a possible prognostic CV power of this SNP, only limited evidence is available so far. Hamann et al. were able to prove an influence of this SNP on restenosis and recommended the inclusion of the SNP in the individual CV profile for risk stratifying as well as for preventive and therapeutic measures [44]. In the present study, however, the influence of this genetic factor on the CV outcome could not be confirmed. The reason for these differences could be found in the follow-up period, which was only 6 months in the study by Hamann et al. and 3 years in the present study. On the other hand, different CV endpoints were referred to (restenosis; present study: CV death, death from stroke, MI, and stroke/TIA).

The TLR4 SNP rs4986790 has been implicated in attenuation of receptor signaling and diminishing the inflammatory response to Gram-negative pathogens. Therefore, its impact on inflammatory diseases including CV diseases was assessed in different studies [23, 45, 46]. But here, as well, the study evidence is very heterogeneous. In an early study by Kiechl et al., investigating 810 individuals, an association of this SNP with CV disease was shown [45]. By contrast, Morange et al. demonstrated that this SNP is not a significant predictor of the CV risk in healthy individuals [46]. These data are in line with the results of two meta-analyses based on 12 and 20 case-control studies, in which nonassociation of this SNP with CV diseases was proven [23, 47]. And also, in the present study, no correlation between this genetic variant and the occurrence of preexisting CV diseases could be shown. Furthermore, SNP rs4986790 in the TLR4 gene has not been associated with cardiovascular prognosis in this study cohort. This result is in line with early studies which demonstrated that in patients with documented CV disease, this SNP was no predictor of future CV events [48] and was not associated with an increased risk of target vessel revascularization or angiographic restenosis after percutaneous coronary intervention [49].

### 4.3. Limitations of the Study

The presented study was performed as a longitudinal cohort study. It was conducted to establish assumptions of the possible prognostic value of genetic variants of CD14 (rs2569190), NF-*κΒ* (rs28362491), TLR2 (rs5743708), and TLR4 (rs4986790) on adverse CV events during a 3-year follow-up period. Considering the study design, the verification of these assumptions is not realizable. The results of the current study are representative for coronary patients in Central Germany and cannot be generally extrapolated to the overall population or other patient cohorts. Furthermore, it is advisable to verify the study results in an additional study.

In the 3-year follow-up, the combined CV endpoint (stroke/TIA, myocardial infarction, CV death, and death from stroke) was investigated. These data were obtained from existing medical records or from civil record offices or by interviewing patients or their relatives (standardized questionnaire, telephone interview). Thus, it is possible that false information from patients or their relatives cannot be excluded due to possible personal incorrect interpretations of the state of health.

## 5. Conclusions

1. At baseline, SNPs in TLR2 (rs5743708), TLR4 (rs4986790), CD14 (rs2569190), and NF-*κΒ* (rs28362491) were not associated with the occurrence of severe periodontitis in CV patients
2. In the 3-year follow-up, the CT genotype of CD14 SNP rs2569190 was proven to be an independent prognostic marker for further cardiovascular events, especially cardiovascular death, considering classical cardiovascular risk factors. All other genetic variants included in the study (NF-*κΒ* (rs28362491), TLR2 (rs5743708), and TLR4 (rs4986790)) had no predictive cardiovascular value

Confirmation of the prognostic value of the CD14 SNP rs2569190 for CV disease should be obtained in further studies. Potentially, this SNP could complement the individual CV risk profile. Integration of stable genetic markers in the CV risk profile assists in the identification of higher risk patients and supports the improvement of individualized therapy.

## Figures and Tables

**Table 1:** Genotyping methods applied for SNPs in CD14, NF-*κΒ*, TLR2, and TLR4.

Gene	SNP	Primer 5′➔3′	Reference sequence	Restriction enzyme
CD14	rs2569190	Forward: gtg cca aca gat gag gtt cac Reverse: cct cct ctg tga acc ctg atc ac	AF097335	HaeIII
NF-*κΒ*	rs28362491	Forward: tgc tgc ctg cgt tcc ccg acc atc g Reverse: ccc gca ggg gcc gcg gcg tcc ag	AF213884S1	Taq*α*I
TLR2	rs5743708	Forward: cat tcc cca gcg ctt ctg caa gct cc Reverse: gga acc tag gac ttt atc gca gct	NM 003264	MspI
TLR4	rs4986790	Forward: gat tag cat act tag acta ct acc tcc atg Reverse: gat caa ctt ctg aaa aag cat tcc cac	NM 138554	NcoI

**Table 2:** Demographical and anamnestic parameters, previous history of diseases, biochemical parameters, and dental parameters of the CV patients.

	CV patients
	*n* = 1002
*Demographical and anamnestic parameters*	
Age (years), median (25^th^/75^th^ IQR)	68.9 (59.8/75)
Male gender (%)	74.0
Current smoking (%)	11.9
Body mass index (kg/m^2^), median (25^th^/75^th^ IQR)	28.1 (25.3/30.9)
*History of*	
Diabetes mellitus (%)	35.7
Hypertension (%)	87.7
MI (%)	38.8
Stroke/TIA (%)	12.7
Peripheral artery disease (%)	9.6
Dyslipoproteinemia (%)	58.8
Severe periodontitis^∗^ (%)	47.4
CV disease known by anamnesis (%)	74.3
CV disease family history (%)	39.8
*Biochemical parameters*	
C-Reactive protein (mg/l), median (25^th^/75^th^ IQR)	8.7 (3.4/31.6)
Leukocytes (Gpt/l), median (25^th^/75^th^ IQR)	7.8 (6.4/9.7)
Interleukin 6 (pg/ml), median (25^th^/75^th^ IQR)	7.4 (3.7/15.8)
Creatinine (mmol/l), median (25^th^/75^th^ IQR)	87 (73/107)
Total cholesterol (mmol/l), median (25^th^/75^th^ IQR)	4.3 (3.7/5.3)
HDL cholesterol (mmol/l), median (25^th^/75^th^ IQR)	1.0 (0.8/1.2)
LDL cholesterol (mmol/l), median (25^th^/75^th^ IQR)	2.6 (2.0/3.3)
Triglycerides (mmol/l), median (25^th^/75^th^ IQR)	1.4 (1.0/1.9)
*Periodontal parameters*	
Plaque index, median (25^th^/75^th^ IQR)	0.8 (0.5/1.4)
Clinical attachment loss (mm), median (25^th^/75^th^ IQR)	4 (3.2/5.1)
Bleeding on probing (%), median (25^th^/75^th^ IQR)	5.6 (1.9/12.5)
Missing teeth (except 8^th^) (*n*), median (25^th^/75^th^ IQR)	11.0 (5.0/19.0)

^∗^Approximal attachment loss of ≥5 mm in ≥30% of the teeth present. Continuous variables were presented as median (25^th^/75^th^ interquartiles (IQR)); TIA: transient ischemic attack; MI: myocardial infarction.

**Table 3:** Association of genotype and allele distribution of SNPs in CD14, NF-*κΒ*, TLR2, and TLR4 and periodontal disease severity.

	All patients	No or mild periodontitis	Severe periodontitis	*p* value
CD14: rs2569190				
	*n* = 928	*n* = 487	*n* = 441	
CC (%)	30.4	29.8	31.1	0.182
CT (%)	46.6	44.8	48.5	
TT (%)	23.0	25.4	20.4	
	*n* = 1856	*n* = 974	*n* = 882	
C (%)	53.7	52.2	55.3	0.186
T (%)	46.3	47.8	44.7	

NF-*κΒ*: rs28362491				
	*n* = 927	*n* = 487	*n* = 440	
II (%)	33.0	31.8	34.3	0.581
ID (%)	51.4	51.5	51.1	
DD (%)	15.6	16.6	14.6	
	*n* = 1854	*n* = 974	*n* = 880	
I (%)	58.7	57.6	59.9	0.341
D (%)	41.3	42.4	40.1	

TLR2: rs5743708				
	*n* = 939	*n* = 493	*n* = 446	
AA (%)	94.5	93.7	95.3	0.389^∗^
AG (%)	5.4	6.1	4.7	
GG (%)	0.1	0.2	0	
	*n* = 1878	*n* = 986	*n* = 892	
A (%)	97.2	96.8	97.6	0.305
G (%)	2.8	3.2	2.4	

TLR4: rs4986790				
	*n* = 942	*n* = 494	*n* = 448	
AA (%)	91.4	92.7	90.0	0.237^∗^
AG (%)	8.4	7.1	9.8	
GG (%)	0.2	0.2	0.2	
	*n* = 1884	*n* = 988	*n* = 896	
A (%)	95.6	96.3	94.9	0.175
G (%)	4.4	3.7	5.1	

^∗^Fisher's exact test.

**Table 4:** Association of genotype and allele distribution of SNPs in CD14. NF-*κΒ*. TLR2 and TLR4 and occurrence of combined endpoint (stroke/TIA. myocardial infarction. Cardiovascular death. Death from stroke).

	All patients	No or mild periodontitis	Severe periodontitis	*p* value
CD14: rs2569190				
	*n* = 881	*n* = 740	*n* = 141	
CC (%)	30.6	31.9	24.1	0.074
CT (%)	46.2	44.6	54.6	
TT (%)	23.2	23.5	21.3	
	*n* = 1762	*n* = 1480	*n* = 282	
C (%)	53.7	54.2	51.4	0.429
T (%)	46.3	45.8	48.6	

NF-*κΒ*: rs28362491				
	*n* = 880	*n* = 738	*n* = 142	
II (%)	32.7	33.5	28.9	0.537
ID (%)	51.4	50.9	53.5	
DD (%)	15.9	15.6	17.6	
	*n* = 1760	*n* = 1476	*n* = 284	
I (%)	58.4	58.9	55.6	0.332
D (%)	41.6	41.1	44.4	

TLR2: rs5743708				
	*n* = 892	*n* = 749	*n* = 143	
AA (%)	94.5	94.4	95.1	1.000^∗^
AG (%)	5.4	5.5	4.9	
GG (%)	0.1	0.1	0	
	*n* = 1784	*n* = 1498	*n* = 286	
A (%)	97.2	97.1	97.6	0.840
G (%)	2.8	2.9	2.4	

TLR4: rs4986790				
	*n* = 895	*n* = 751	*n* = 144	
AA (%)	91.2	91.5	89.6	0.590^∗^
AG (%)	8.6	8.3	10.4	
GG (%)	0.2	0.2	0.0	
	*n* = 1790	*n* = 1502	*n* = 288	
A (%)	95.5	95.6	94.8	0.650
G (%)	4.5	4.4	5.2	

^∗^Fisher's exact test.

**Table 5:** Cox regression analysis evaluating the hazard ratio (HR) of the CT genotype of CD14 SNP rs2569190 for the incidence of the (a) combined endpoint (stroke/TIA, myocardial infarction, cardiovascular death, and death from stroke) and (b) cardiovascular death within a 3-year follow-up period (adjusted for age, gender, smoking, body mass index, hypertension, hyperlipoproteinemia, diabetes, and severe periodontitis).

Confounding variables	Hazard ratio	95% lower	CI upper	P values
(a) Endpoint: stroke/TIA, myocardial infarction, cardiovascular death, and death from stroke				
*CT genotype rs2569190*	*1.572*	*1.012*	*2.439*	*0.044*
Age	1.063	1.035	1.092	<0.001
Diabetes	2.366	1.501	3.729	<0.001
Male gender	1.302	0.784	2.162	0.309
Current smoking	1.572	0.469	5.262	0.463
Hypertension	1. 413	0.706	2.828	0.329
Hyperlipoproteinemia	1.037	0.657	1.636	0.876
Severe periodontitis	1.121	0.721	1.742	0.613
(b) Endpoint: cardiovascular death				
*CT genotype rs2569190*	*1.585*	*1.020*	*2.500*	*0.040*
Age	1.063	1.035	1.092	<0.001
Diabetes	2.366	1.501	3.729	<0.001
Male gender	1.280	0.772	2.125	0.339
Current smoking	1.563	0.467	5.233	0.469
Hypertension	1. 411	0.705	2.825	0.331
Hyperlipoproteinemia	1.052	0.6668	1.658	0.826
Severe periodontitis	1.116	0.717	1.736	0.626

## Data Availability

All data are available on request from the corresponding author (Susanne Schulz, Martin Luther University Halle-Wittenberg, Medical Faculty, Department of Operative Dentistry and Periodontology, Magdeburger Str. 16, 06112 Halle (Saale), Germany).
